# Left atrial intramyocardial fat at pulmonary vein reconnection sites during atrial fibrillation redo ablation

**DOI:** 10.1093/europace/euaf038

**Published:** 2025-02-20

**Authors:** Federico Landra, Andrea Saglietto, Giulio Falasconi, Diego Penela, David Soto-Iglesias, Emanuele Curti, Bruno Tonello, Lucio Teresi, Dario Turturiello, Paula Franco-Ocaña, Carlo Gigante, Chiara Valeriano, Claudio Capobianco, Pietro Francia, José Alderete, Daniel Viveros, Aldo Francisco Bellido, Fatima Zaraket, Julio Martí-Almor, Matteo Cameli, Antonio Berruezo

**Affiliations:** Arrhythmia Department, Teknon Heart Institute, Teknon Medical Center, C/Vilana 12, Barcelona 08022, Spain; Department of Medical Biotechnologies, Division of Cardiology, University of Siena, Siena, Italy; Arrhythmia Department, Teknon Heart Institute, Teknon Medical Center, C/Vilana 12, Barcelona 08022, Spain; Division of Cardiology, Cardiovascular and Thoracic Department, ‘Citta della Salute e della Scienza’ Hospital, Turin, Italy; Department of Medical Sciences, University of Turin, Turin, Italy; Arrhythmia Department, Teknon Heart Institute, Teknon Medical Center, C/Vilana 12, Barcelona 08022, Spain; Campus Clínic, University of Barcelona, Barcelona, Spain; Arrhythmia Department, Teknon Heart Institute, Teknon Medical Center, C/Vilana 12, Barcelona 08022, Spain; Electrophysiology Department, IRCCS Humanitas Research Hospital, Rozzano, Milan, Italy; Arrhythmia Department, Teknon Heart Institute, Teknon Medical Center, C/Vilana 12, Barcelona 08022, Spain; Arrhythmia Department, Teknon Heart Institute, Teknon Medical Center, C/Vilana 12, Barcelona 08022, Spain; Department of Medicine and Surgery, University of Milano-Bicocca, Milan, Italy; Arrhythmia Department, Teknon Heart Institute, Teknon Medical Center, C/Vilana 12, Barcelona 08022, Spain; Arrhythmia Department, Teknon Heart Institute, Teknon Medical Center, C/Vilana 12, Barcelona 08022, Spain; Department of Clinical and Experimental Medicine, University of Messina, Messina 98100, Italy; Arrhythmia Department, Teknon Heart Institute, Teknon Medical Center, C/Vilana 12, Barcelona 08022, Spain; Arrhythmia Department, Teknon Heart Institute, Teknon Medical Center, C/Vilana 12, Barcelona 08022, Spain; Arrhythmia Department, Teknon Heart Institute, Teknon Medical Center, C/Vilana 12, Barcelona 08022, Spain; Division of University Cardiology, IRCCS Ospedale Galeazzi-Sant'Ambrogio, Milan, Italy; Arrhythmia Department, Teknon Heart Institute, Teknon Medical Center, C/Vilana 12, Barcelona 08022, Spain; Department of Advanced Biomedical Sciences, University of Naples Federico II, Naples, Italy; Arrhythmia Department, Teknon Heart Institute, Teknon Medical Center, C/Vilana 12, Barcelona 08022, Spain; Arrhythmia Department, Teknon Heart Institute, Teknon Medical Center, C/Vilana 12, Barcelona 08022, Spain; Department of Clinical and Molecular Medicine, Cardiology Unit, Sant’Andrea Hospital, University Sapienza, Rome, Italy; Arrhythmia Department, Teknon Heart Institute, Teknon Medical Center, C/Vilana 12, Barcelona 08022, Spain; Arrhythmia Department, Teknon Heart Institute, Teknon Medical Center, C/Vilana 12, Barcelona 08022, Spain; Arrhythmia Department, Teknon Heart Institute, Teknon Medical Center, C/Vilana 12, Barcelona 08022, Spain; Arrhythmia Department, Teknon Heart Institute, Teknon Medical Center, C/Vilana 12, Barcelona 08022, Spain; Arrhythmia Department, Teknon Heart Institute, Teknon Medical Center, C/Vilana 12, Barcelona 08022, Spain; Department of Medical Biotechnologies, Division of Cardiology, University of Siena, Siena, Italy; Arrhythmia Department, Teknon Heart Institute, Teknon Medical Center, C/Vilana 12, Barcelona 08022, Spain

**Keywords:** Catheter ablation, Atrial fibrillation, Redo ablation, Intramyocardial fat, Pulmonary vein reconnection

## Abstract

**Aims:**

Electrical reconnections between pulmonary veins (PVs) and the left atrium (LA) are frequently responsible for atrial fibrillation (AF) recurrences after pulmonary vein isolation (PVI). Multidetector computed tomography (MDCT)-derived images can be post-processed to detect intramyocardial fat (inFAT) by signal radiodensity thresholding. The role of inFAT on PV-LA reconnections remains unknown. The aim of this study was to analyse the relationship between inFAT localization at pre-procedural MDCT-derived inFAT maps from first AF ablation and PV-LA reconnections in patients with AF recurrence undergoing redo ablation.

**Methods and results:**

We included 45 consecutive patients who underwent AF redo ablation presenting at least one PV-LA reconnection. First AF ablation pre-procedural MDCT-derived data were post-processed with ADAS 3D™ to create 3D LA inFAT maps, which were loaded into CARTO3 navigation system and merged with the electroanatomical map for reconnection site analysis. In 103 out of 156 (66.0%), PV-LA reconnection points inFAT was identified in the 6 mm diameter tag point depicted in the navigation system. When dividing the PVI line into standardized segments, those identified as containing PV-LA reconnection points (i.e. reconnection segments) exhibited significantly higher total inFAT volumes compared with non-reconnection segments (8.05 ± 6.56 vs. 5.40 ± 5.18 μL, *P* < 0.001). Additionally, reconnection segments showed greater volumes of inFAT components, specifically dense inFAT (0.06 ± 0.06 vs. 0.03 ± 0.04 μL, *P* < 0.001) and fat-myocardial admixture (7.98 ± 6.52 vs. 5.37 ± 5.16 μL, *P* < 0.001).

**Conclusion:**

Intramyocardial fat is co-localized with two-thirds of PV-LA reconnection points in patients undergoing AF redo ablation. Reconnection segments exhibit significantly higher inFAT volumes compared to non-reconnection segments. This proof-of-concept study suggests that inFAT may play a role in PV-LA electrical reconnections following PVI.

## Graphical Abstract

**Graphical Abstract:**
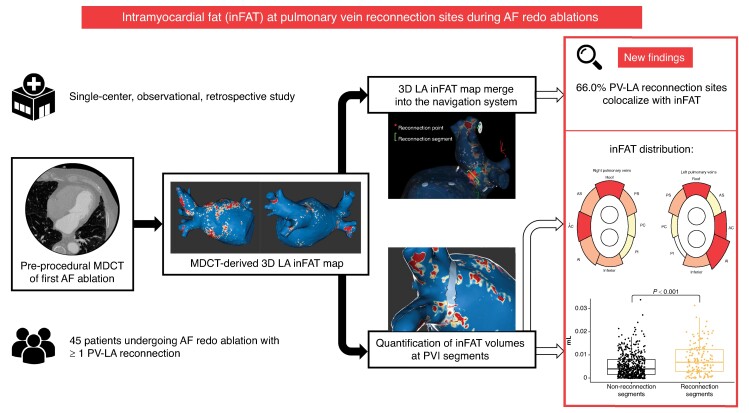
InFAT, intramyocardial fat; AF, atrial fibrillation; PV, pulmonary vein; LA, left atrium; MDCT, multidetector computed tomography; PVI, pulmonary vein isolation; AS, antero-superior segment; AC, antero-carinal segment; AI, antero-inferior segment; PI, postero-inferior segment; PC, postero-carinal segment; PS, postero-superior segment.

**What’s new?**

- In patients undergoing AF redo ablation procedures, first AF ablation pre-procedural MDCT-derived intramyocardial fat (inFAT) colocalize with two-thirds of PV-LA reconnection sites.
- When dividing the pulmonary vein isolation (PVI) line into standardized segments, reconnection segments exhibit higher inFAT volumes than non-reconnection segments, including threshold subranges dense inFAT and fat-myocardial admixture.
- Intramyocardial fat may explain part of the PV-LA late electrical reconnections after PVI because of its biophysical properties and histological architecture.

## Introduction

Atrial fibrillation (AF) is the most frequent cardiac arrhythmia in adults and radiofrequency (RF) catheter ablation is an established and widely adopted strategy for rhythm control.^1^ Pulmonary vein isolation (PVI) is the cornerstone of catheter-based strategies for AF ablation.^1^ Despite the significant technical and technological improvements introduced in the last decade, delivering transmural durable lesions remains a main issue of PVI.^2^ Even though acute procedural success is now achievable in a high percentage of cases, late electrical reconnections between pulmonary veins (PVs) and the left atrium (LA) are frequently associated with arrhythmic recurrences.^3–6^

Previous studies demonstrated that PV-LA reconnections are more frequently observed in thicker segments of the PVI line.^7^ A personalized PVI approach using ablation index titration according to the local left atrial wall thickness (LAWT) as assessed with multidetector cardiac tomography (MDCT)-data post-processing should ideally overcome the transmurality dilemma.^8,9^ However, thickness is only one anatomical feature of a complex three-dimensional (3D) matrix that may harbour other histological components apart from myocardial tissue, such as fibrosis and adipose tissue among others.^1,10,11^

Intramyocardial fat (inFAT) may host branches of the autonomic nervous system and, similarly to fibrosis, may favour electrophysiological phenomena such as endo-epicardial dissociation and breakthrough phenomena.^1,12–17^ Compared to atrial myocardium, adipose tissue is characterized by a 3-times and 10-times lower thermal and electrical conductivity, respectively, with potential consequences on RF delivery and on the formation of irreversible lesions.^18,19^ MDCT-derived images can be post-processed to detect inFAT by signal density thresholding.^10,20^

The aim of this study was to analyse the relationship between inFAT localization at pre-procedural MDCT-derived inFAT maps from first AF ablation and PV-LA reconnections in patients with AF recurrence undergoing redo ablation.

## Methods

### Patient sample

This was a single-centre, observational, retrospective, proof-of-concept study. Consecutive patients who underwent first AF redo ablation between February 2023 and February 2024 were screened for possible inclusion in the study. Inclusion criteria were ≥18 years of age, having ≥1 PV-LA reconnections at AF redo ablation, and an available pre-procedural MDCT scan of the first AF ablation with adequate image quality. Paroxysmal and persistent AF was defined according to the definition of the latest European Society of Cardiology (ESC) guidelines.^21^ All patients had documented recurrences of symptomatic AF and indication for ablation in accordance with ESC guidelines.

All patients provided written informed consent before the procedure. The study was reviewed and approved by the local ethics committee, follows the STROBE guidelines for observational studies, and complied with the Declaration of Helsinki.

### MDCT scan and image post-processing

Pre-procedural MDCT was performed with a Revolution™ CT scanner (General Electric Healthcare). The images were acquired during an inspiratory breath-hold using retrospective ECG-gating technique with tube current modulation set between 50% and 100% of the cardiac cycle. MDCT data were analysed with ADAS 3D™ software (Galgo Medical, Barcelona, Spain) to obtain 3D LAWT maps, as previously described.^7,10,22,23^ Image post-processing for 3D LAWT maps was performed blinded to the PV-LA reconnection sites’ location. 3D inFAT maps were generated using a threshold-based segmentation of the volume between the endocardial and the epicardial LA shells previously obtained for 3D LAWT maps. Intramyocardial fat was defined as a tissue with reduced radiodensity, in the range of −194 Hounsfield units (HU) to −5 HU. In addition, two specific inFAT subranges were explored, the former between −194 and −50 HU (dense inFAT) and the latter between −50 and −5 HU (fat-myocardium admixture).^24,25^ LA appendage and the more distal segments of the PVs (>5 mm from the ostia) were excluded from the segmentation for 3D inFAT map rendering, as previously described.^10^

**Figure 1:**
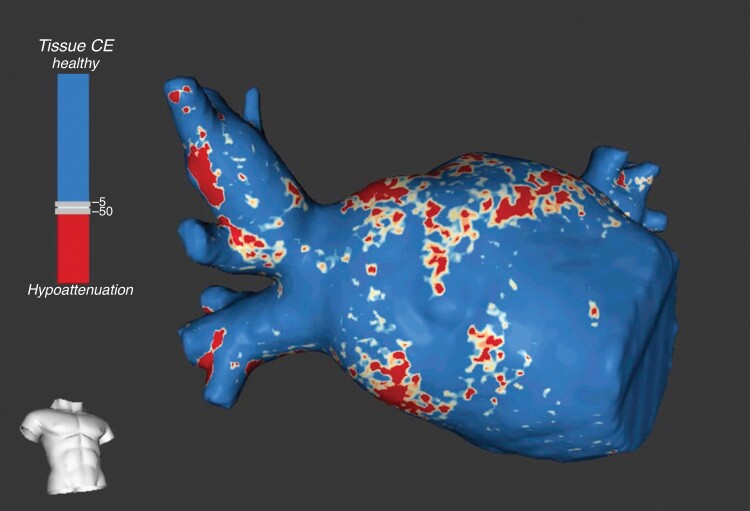
MDCT-derived 3D inFAT map. Septal view of a LA 3D inFAT map. Red areas correspond to dense inFAT (−194 to −50 HU), yellow-orange areas to fat-myocardial admixture (−50 to −5 HU), and blue areas do not harbour inFAT at all (>−5 HU). MDCT, multidetector computed tomography; inFAT, intramyocardial fat; LA, left atrial; HU, Hounsfield unit.

*Figure 1* represents a MDCT-derived LA inFAT 3D map rendering.

### Redo ablation procedure and PV-LA reconnection site identification

All procedures were performed using CARTO3 non-fluoroscopic mapping system (Biosense Webster, Johnson & Johnson Medical S.p.A., CA, USA). Transseptal puncture was guided by transoesophageal echocardiography, performed by cardiologists experienced in cardiac imaging.^26^ All AF redo ablation procedures were performed with single catheter technique,^7,27^ using a contact force-sensing irrigated ablation catheter (Thermocool Smarttouch, Biosense Webster Inc., CA, USA). A fast-anatomical map of the entire LA anatomy and the PVs was acquired and then merged with the imported 3D LAWT map within the spatial reference co-ordinates of the CARTO system, as part of the standard approach in our centre.^7^

PV reconnection was defined by the absence of entrance and/or exit block. In such cases, PV-LA reconnection sites were looked after by analysing electrograms on the EP recording system (EP-TRACER, Schwarzer Cardiotek, Heilbronn, Germany) recorded with the ablation catheter according to a systematic, standardized local approach. For this purpose, the EP recording was set in trigger mode. The ablation catheter was sequentially moved all way through the expected prior circumferential PVI line. The earliest PV potential with respect to the reference calliper was considered the reconnection site. If electrical activation inside the vein was still observed after ablation of the first designated PV-LA reconnection point, the mapping process was repeated until all the PV-LA reconnected points were identified. Each one of these points was considered as a PV-LA reconnection site and included in the analysis. A distance of >5 mm from the prior circumferential PVI line presenting the earliest PV electrogram defined epicardial connections, which were not included in the present analysis.^28^ During the ablation procedure, each identified PV-LA reconnection site was marked with a tag point (6 mm diameter). Radiofrequency was applied at the PV-LA reconnection sites until entrance and exit block at all PVs was achieved. Ablation parameters were 35–50 W, 45°C, with ablation index adapted to the local LAWT, as previously described.^29^ The acute procedural endpoint was PVI, which was confirmed by entry block with absence of PV potentials inside the vein with the ablation catheter placed sequentially in each segment inside the circumferential PVI line, and exit block by proving absence of atrial capture when pacing (10 mA, 2 ms) from inside the circumferential PVI line sequentially at each segment.^27^

### PV-LA reconnection site analysis

Firstly, 3D LA inFAT maps derived from pre-procedural MDCT scan of the first AF ablation were imported into CARTO system and aligned according to AF redo ablation spatial co-ordinates for PV-LA reconnection site analysis. Tag points generated during AF redo ablation procedures corresponding to PV-LA reconnection sites were used for PV-LA reconnection site analysis. Intramyocardial fat presence was assessed at each PV-LA reconnection site previously tagged and reported in a dedicated database. Secondly, 3D inFAT maps derived from first AF ablation MDCT scans were used to quantify total inFAT, dense inFAT, and fat-myocardium admixture volumes at each PVI segment (roof, antero-superior, antero-carinal, antero-inferior, inferior, posterior-inferior, posterior-carinal, posterior-superior segments). Reconnection segments were defined as PVI segments containing ≥1 PV-LA reconnection point. Intramyocardial fat volumes at reconnection segments were subsequently compared to inFAT volumes at non-reconnection segments.

**Figure 2:**
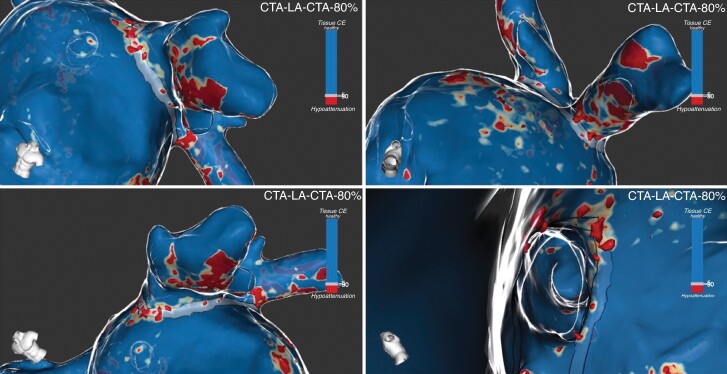
PVI line analysis. MDCT-derived 3D inFAT maps of the LA from different perspectives. A 6 mm wide band corresponding to PVI line trajectory was traced. Top-left panel: lateral–cranial view of the left PVI line; top-right panel: lateral–caudal view of the left PVI line; bottom-left panel: lateral–cranial view of the left PVI line; bottom-right panel: internal medial-to-lateral view of the left PVI line. MDCT, multidetector computed tomography; inFAT, intramyocardial fat; LA, left atrial; PVI, pulmonary vein isolation.

*Figure 2* represents MDCT-derived LA inFAT 3D map renderings and inFAT presence at PVI line.

**Figure 3:**
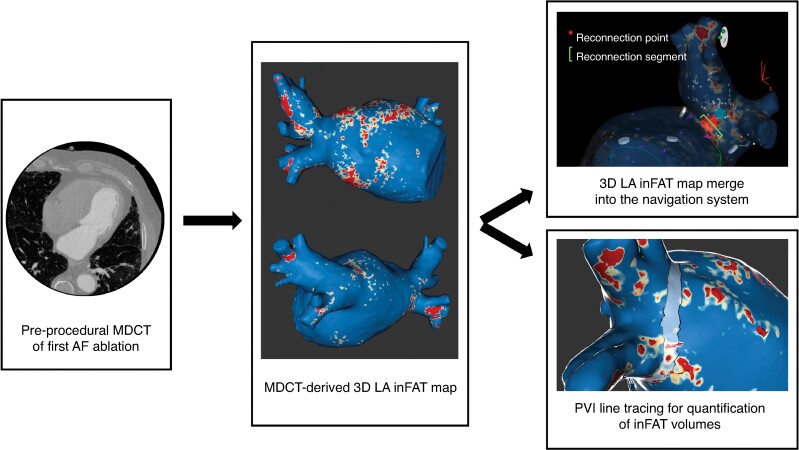
Procedural workflow. Pre-procedural MDCT scan of first AF ablations were post-processed. 3D inFAT maps were imported into the navigation system for PV-LA reconnection site analysis (identification of spatial colocalization between PV-LA reconnection points and inFAT). Also, PVI line was traced on 3D inFAT maps with the aim of quantifying the inFAT volume at each PVI line segment (total inFAT, dense inFAT, and fat-myocardial admixture). MDCT, multidetector computed tomography; AF, atrial fibrillation; LA, left atrium; inFAT, intramyocardial fat; PVI, pulmonary vein isolation.

*Figure 3* represents the procedural workflow of the MDCT-derived 3D inFAT maps rendering and analysis.

##  

### Statistical analysis

Continuous data are presented as mean and standard deviation or as median and interquartile range, as appropriate. Kolmogorov–Smirnov test was used to verify normal distribution of variables. Categorical data are summarized as absolute and relative frequencies. Continuous variables were compared using the unpaired *t*-test for normally distributed variables and the nonparametric Mann–Whitney *U* test for non-normally distributed variables. The χ^2^ test was used for categorical variables. MATLAB statistics toolbox (Matlab R2020a, The Mathworks, Inc., Natick, MA, USA) was used for advanced analysis on inFAT volumes at PVI segments using a customized code. In case of missing data, no statistical method for imputation was performed. A *P*-value of <0.05 was considered statistically significant. Analysis was performed using SPSS, version 26 (SPSS, Chicago, IL, USA), and R Statistical Software, version 3.6.3 (R Foundation for Statistical Computing, Vienna, Austria).

## Results

### Patient population

According to inclusion criteria, 45 patients who underwent an AF redo ablation procedure between February 2023 and February 2024 at the study centre had ≥1 PV-LA reconnections and were therefore included. All patients had an available pre-procedural MDCT scan of the first AF ablation, and MDCT image quality was deemed sufficient for inFAT segmentation in all of them. Patients’ baseline characteristics are resumed in *Table 1*. Twenty-three (51.1%) patients were male, the mean age was 63 ± 11 years, and the mean LA volume was 75.7 ± 54.1 mL. The majority of arrhythmic recurrences was paroxysmal AF, observed in 30 patients (66.7%), followed by persistent AF, which occurred in 15 patients (33.3%).

**Table 1:** Baseline characteristics

Variables	All patients (*n* = 45)
Age (years)	63 ± 11
Males (%)	23 (51.1)
Weight (kg)	82 ± 16
Height (cm)	172 ± 9
Hypertension (%)	20 (44.4)
Dyslipidaemia (%)	13 (28.9)
Smoke (%)	3 (6.7)
Diabetes (%)	1 (2.2)
CHA2DS2-VASc	2.0 [0.5–3.0]
Underlying heart disease	
None	40 (88.9)
Hypertensive	1 (2.2)
Ischaemic	0 (0)
Hypertrophic	1 (2.2)
Valvular	2 (4.4)
Dilative	0 (0)
Other	1 (2.2)
Anticoagulation (%)	39 (86.7)
AAD	
Flecainide	14 (31.1)
Amiodarone	14 (31.1)
Propafenone	0 (0)
Sotalol	2 (4.4)
Betablockers	21 (46.7)
LA dimension (mm)	41.6 ± 7.7
LA volume (mm^3^)	75.7 ± 54.1
PV anatomy	
4 independent PVs	38 (84.4)
LCPV	6 (13.3)
RCPV	0 (0)
LCPV + RCPV	0 (0)
Intermediate RPV	1 (2.2)
LVEF (%)	60 [60–65]
Mitral regurgitation	
Mild	11 (24.4)
Moderate	2 (4.4)
Severe	1 (2.2)
AF classification at first ablation	
Paroxystic	30 (66.7)
Persistent	15 (33.3)
Days between redo and first ablation	422 [292–716]

AAD, antiarrhythmic drug; LA, left atrium; PV, pulmonary vein; LCPV, left common pulmonary vein; RCPV, right common pulmonary vein; RPV, right pulmonary vein; LVEF, left ventricular ejection fraction; AF, atrial fibrillation.

### Redo ablation procedures

A median of 422 [292–716] days passed between the first ablation procedures and the redo procedures. Overall, 157 reconnection points were identified at AF redo procedures, with a median number of PV-LA reconnection points/patients of 3.0 [2.0–5.0]. One (0.6%) of reconnection point was excluded because involving an epicardial connection.

PV-LA reconnection points at right PVs were identified in the following segments: 12 (14.1%) at the superior segment, 16 (18.8%) at the antero-superior segment, 22 (25.9%) at the antero-carinal segment, 11 (12.9%) at the antero-inferior segment, 6 (7.1%) at the inferior segment, 4 (4.7%) at the posterior-inferior segment, 9 (10.6%) at the posterior-carinal segment, and 5 (5.9%) at the posterior-superior segment, for a total of 85 PV-LA reconnection points at right PVs.

PV-LA reconnection points at left PVs were identified in the following segments: 5 (7.0%) at the superior segment, 20 (28.2%) at the antero-superior segment, 16 (22.5%) at the antero-carinal segment, 16 (22.5%) at the antero-inferior segment, 3 (4.2%) at the inferior segment, 6 (8.5%) at the posterior-inferior segment, 2 (2.8%) at the posterior-carinal segment, and 3 (4.2%) at the posterior-superior segment, for a total of 71 PV-LA reconnection points at left PVs.

### Intramyocardial fat at first ablation MDCT-derived map

**Figure 4:**
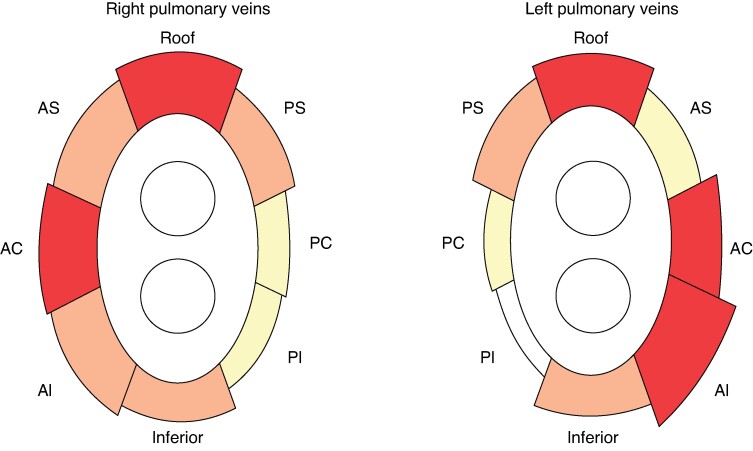
Intramyocardial fat distribution at PVI line segments. First ablation MDCT-derived total inFAT volume distribution at various PVI line segments is depicted in a proportional manner (red ≥ 7.00 μL; orange ≥ 5.00 μL, yellow ≥ 3.00 μL, white < 3.00 μL). Both right PVs and left PVs show a higher amount of total inFAT at roof, antero-carinal, and antero-inferior segments. InFAT, intramyocardial fat; PVI, pulmonary vein isolation; MDCT, multidetector computed tomography; PV, pulmonary vein; AS, antero-superior segment; AC, antero-carinal segment; AI, antero-inferior segment; PI, postero-inferior segment; PC, postero-carinal segment; PS, postero-superior segment.

*Figure 4* and *Table 2* represent inFAT volume distribution at PVI line segments at pre-procedural MDCT segmentation of first AF ablation procedure.

**Table 2:** Intramyocardial fat distribution at PVI line segments at first ablation MDCT segmentation

PVI segments	Total inFAT (μL)	Dense inFAT (μL)	Fat-myocardial admixture (μL)	Number of PV-LA reconnections (*n*, %)
Right PVs				
Roof	8.41 ± 6.09	0.06 ± 0.06	8.36 ± 6.05	12 (14.1)
AS	6.82 ± 4.96	0.04 ± 0.06	6.78 ± 4.92	16 (18.8)
AC	7.54 ± 6.85	0.05 ± 0.06	7.49 ± 6.81	22 (25.9)
AI	6.95 ± 4.37	0.05 ± 0.06	6.90 ± 4.34	11 (12.9)
Inferior	5.84 ± 5.36	0.04 ± 0.05	5.80 ± 5.33	6 (7.1)
PI	3.49 ± 4.21	0.02 ± 0.03	3.47 ± 4.19	4 (4.7)
PC	4.35 ± 4.49	0.03 ± 0.04	4.33 ± 4.46	9 (10.6)
PS	5.84 ± 5.59	0.04 ± 0.05	5.81 ± 5.56	5 (5.9)
Left PVs				
Roof	7.44 ± 7.27	0.05 ± 0.06	7.40 ± 7.22	5 (7.0)
AS	4.56 ± 4.47	0.02 ± 0.03	4.54 ± 4.44	20 (28.2)
AC	7.10 ± 5.28	0.04 ± 0.04	7.07 ± 5.25	16 (22.5)
AI	9.41 ± 6.63	0.04 ± 0.04	9.37 ± 6.61	16 (22.5)
Inferior	6.29 ± 5.33	0.03 ± 0.03	6.26 ± 5.31	3 (4.2)
PI	2.43 ± 3.84	0.01 ± 0.02	2.42 ± 3.82	6 (8.5)
PC	3.33 ± 3.67	0.02 ± 0.03	3.32 ± 3.65	2 (2.8)
PS	5.75 ± 5.71	0.03 ± 0.06	5.72 ± 5.67	3 (4.2)

PVI, pulmonary vein isolation; inFAT, intramyocardial fat; PV, pulmonary vein; LA, left atrium; AS, antero-superior; AC, antero-carinal; AI, antero-inferior; PI, postero-inferior; PC, postero-carinal; PS, postero-superior.

3D InFAT maps derived from first ablation pre-procedural MDCT scans of the included patients showed the highest amount of total inFAT volume at roof (8.41 ± 6.09 μL), antero-carinal segment (7.54 ± 6.85 μL), and antero-inferior segment (6.95 ± 4.37 μL) of right PVs, and at antero-inferior segment (9.41 ± 6.63 μL), roof (7.44 ± 7.27 μL), and antero-carinal segment (7.10 ± 5.28 μL) of left PVs.

### Reconnection site analysis

In 103 over 156 PV-LA reconnection points (66.0%) identified at AF redo ablation, inFAT was identified in the 6 mm diameter tag point depicted in the CARTO3 navigation system. Total inFAT volume at reconnection segments was significantly higher than at non-reconnection segments (8.05 ± 6.56 μL vs. 5.40 ± 5.18 μL, *P* < 0.001) (*Graphical Abstract*). Similarly, dense inFAT and fat-myocardial admixture volumes at reconnection segments were significantly higher than at non-reconnection segments (0.06 ± 0.06 μL vs. 0.03 ± 0.04 μL, *P* < 0.001 for dense inFAT; 7.98 ± 6.52 μL vs. 5.37 ± 5.16 μL, *P* < 0.001 for fat-myocardium admixture).

**Figure 5:**
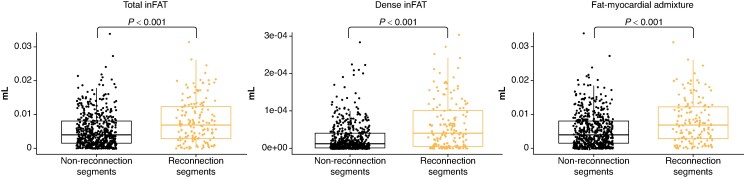
Total inFAT, dense inFAT, and fat-myocardial admixture volumes dot plots. Intramyocardial fat volumes comparison between reconnection segments and non-reconnection segments. Reconnection segments have significantly higher total inFAT, dense inFAT, and fat-myocardial admixture volumes compared to non-reconnection segments. InFAT, intramyocardial fat.

*Figure 5* represents dot plots comparing total inFAT, dense inFAT, and fat-myocardial admixture volumes in reconnection segments vs. non-reconnection segments.

## Discussion

To our knowledge, this is the first study to examine the relationship between inFAT and PV-LA reconnection sites during AF redo ablation procedures. The main findings are that MDCT-derived LA inFAT colocalize with two-thirds of PV-LA reconnection sites in patients undergoing AF redo ablation and that reconnection segments exhibit higher inFAT volumes than non-reconnection segments.

### Intramyocardial fat and atrial fibrillation

In a recent study, it has been demonstrated that post-processing of MDCT images of the LA enables the reconstruction of LA inFAT maps and provides an accurate quantification of fat infiltration.^10^ Reproducibility of intramyocardial adipose infiltration detection by MDCT images has been recognized in recently published international consensus documents as well.^1,20^ This previous pioneering study identified four main findings: (i) the degree of fat infiltration in the LA is independent of a patient’s body mass index; (ii) the superior sub-segments of the anterior wall, including the LA roof and septal wall showed the most prominent fatty infiltration; (iii) the level of LA fat infiltration appears to be associated with a higher likelihood of AF, as it was significantly greater in AF patients compared to controls; and (iv) persistent forms of AF are associated with greater degree of inFAT, while control patients are characterized by the lowest inFAT amount. These results suggest that LA inFAT could be a key element in the complex pathophysiological process of AF development and progression, supporting the previously proposed theory by Nalliah *et al*.^17^ that fat infiltration may locally increase atrial conduction heterogeneity, ultimately raising the vulnerability to three-dimensional re-entrant circuits. The results of the present study confirm the initial findings of Saglietto *et al*.,^10^ as we observed significant intramyocardial adipose infiltration into the LA in a different population of AF patients, with particular predisposition to inFAT localization in the roof and septal segments.

### Intramyocardial fat implication in PV-LA reconnections

The results of the present study suggest a significant impact of intramyocardial adipose infiltration around PV ostia on reconnections at PVI line. Notably, two-thirds of PV-LA reconnection sites contained inFAT on initial MDCT segmentation prior to the first AF ablation. Furthermore, reconnection segments exhibited a significantly greater volume of inFAT (both dense inFAT and fat-myocardium admixture) compared to non-reconnection segments. Biophysically, adipose tissue has three times lower thermal conductivity than atrial myocardium,^18,19^ which may hinder adequate energy penetration to achieve transmural and durable lesions. Interestingly, most inFAT along the PVI line is composed by fat-myocardium admixture, where cardiomyocytes intermingle with adipocytes in a heterogeneous, anisotropic matrix. This complex 3D matrix may provide pathways for tortuous activation and delayed conduction, as well as creates a protective niche for embedded cardiomyocytes due to adipose tissue’s high resistivity.^18^

### Clinical perspectives

This study generates the hypothesis that intramyocardial adipose infiltration may protect cardiomyocytes from radiofrequency energy, thereby preventing the formation of durable lesions. Should this hypothesis be confirmed in future studies with a larger patient sample, this would have a great clinical impact from the point of view of patient selection for AF ablation, AF ablation technique, energy source, or the way of energy delivery to isolate PVs. Since this information can be available real-time with the integration of the inFAT maps into the navigation system, further approaches modulating the radiofrequency energy based on the degree of inFAT infiltration could be explored. This observation further supports the spread of pre-procedural imaging to enhance safety and effectiveness of AF ablation.^20^ The findings of this study shed light on a possible pitfall for non-thermal energy sources such as electroporation, since adipose tissue is characterized by 10-times lower electrical conductivity with regard to atrial myocardium.^18,19^ The inFAT preferential distribution in the superior PVs could be an explanation for the highest number of PV-LA reconnections at the anterior aspects of the upper PVs found in patients who previously underwent PVI by means of pulse-field ablation.^30^ Further studies are needed to explore this hypothesis.

### Study limitations

The most important limitation of the study is its retrospective single-centre design; being an observational cross-sectional study, a cause-effect relationship between the presence of inFAT and PV-LA reconnections cannot be demonstrated. Secondly, reconnection site analysis relied on the merging between first ablation MDCT-derived maps and the FAM acquired at AF redo procedure, with potential spatial inaccuracy; however, first ablation MDCT scans were chosen because it cannot be excluded that radiofrequency ablation might have modified the HU of the underlying tissue. Finally, the absence of very-high density electroanatomical maps limited the spatial definition of PV-LA reconnections sites. However, the single catheter approach has previously demonstrated to be a feasible, accurate, and efficient for AF redo procedures.^7,27^

## Conclusion

In patients undergoing AF redo ablation procedures, MDCT-derived inFAT colocalize with two-thirds of PV-LA reconnection sites. Moreover, reconnection segments harbour higher inFAT volumes than non-reconnection segments, including threshold subranges dense inFAT and fat-myocardial admixture. This proof-of-concept study suggests that inFAT may explain part of the PV-LA late electrical reconnections after PVI because of its biophysical properties and histological architecture.

## Authors’ contributions

F.L.: study concept and design, manuscript drafting, critical revision. A.S.: study concept and design, manuscript drafting, critical revision. G.F.: study concept and design, manuscript drafting, critical revision. D.P.: study concept and design, manuscript drafting, critical revision. D.S.-I.: study concept and design, manuscript drafting, critical revision. E.C.: study concept and design, manuscript drafting, critical revision. B.T.: study concept and design, manuscript drafting, critical revision. L.T.: study concept and design, manuscript drafting, critical revision. D.T.: study concept and design, manuscript drafting, critical revision. P.F.-O.: study concept and design, manuscript drafting, critical revision. C.G.: study concept and design, manuscript drafting, critical revision. C.V.: study concept and design, manuscript drafting, critical revision. C.C.: study concept and design, manuscript drafting, critical revision. P.F.: study concept and design, manuscript drafting, critical revision. J.A.: study concept and design, manuscript drafting, critical revision. D.V.: study concept and design, manuscript drafting, critical revision. A.F.B.: study concept and design, manuscript drafting, critical revision. F.Z.: study concept and design, manuscript drafting, critical revision. J.M.-A.: study concept and design, manuscript drafting, critical revision. M.C.: study concept and design, manuscript drafting, critical revision. A.B.: study concept and design, manuscript drafting, critical revision.

## Data Availability

The data underlying this article will be shared on reasonable request to the corresponding author.
